# Trends in *Helicobacter pylori*-related gastric ulcer research from 2012 to 2022: A bibliometric and visual analysis

**DOI:** 10.3389/fmed.2022.1027534

**Published:** 2022-11-23

**Authors:** Chang Yu, Jingyue Qiu, Meng Xiong, Chen Ou, Meiyan Zeng, Houpan Song

**Affiliations:** ^1^Hunan Provincial Key Laboratory of Diagnostic Research in Chinese Medicine, Hunan University of Chinese Medicine, Changsha, Hunan, China; ^2^College of Traditional Chinese Medicine, Hunan University of Chinese Medicine, Changsha, Hunan, China

**Keywords:** gastric ulcer, *Helicobacter pylori*, bibliometric, knowledge maps, VOSviewer, CiteSpace

## Abstract

**Background:**

*Helicobacter pylori*-related gastric ulcer (*H. pylori*-related GU) is one of the most common digestive system diseases that have received widespread attention from researchers. The purpose of this article was to analyze the research status and hotspots of *H. pylori*-related GU and to predict its future research directions.

**Methods:**

The article and review papers associated with *H. pylori*-related GU published from 2012 to 2022 were retrieved from the Web of Science Core Collection (WoSCC). The analysis of knowledge maps and bibliometrics was done with CiteSpace 6.1.R2 Basic and VOSviewer 1.6.18.

**Results:**

A total of 2,971 articles were included in the study. Between 2012 and 2022, the number of papers published showed an increasing trend. China was the most prolific country, and the United States was the most influential country. Baylor College of Medicine had the largest number of publications and citations among publishing agencies. *World Journal of Gastroenterology* published the most articles on the *H. pylori*-related GU field, and *GUT* was the journal with the most cited articles. Yamaoka Y from Japan was the most productive author, and Graham DY from the USA was the most influential author. A keyword and reference analysis showed that the hot topics of research were the mechanism of *H. pylori* and the treatment of *H. pylori*-related GU. The keywords that emerged in the recent 5 years were oxidative stress, probiotics, competitive acid blocker, vonoprazan, gut microbiota, and neutrophil-activating protein.

**Conclusion:**

Over the recent 10 years, research on *H. pylori*-related GU has generally shown an increasing trend. The treatment and pathogenesis of *H. pylori*-related GU remain a hot topic of research. The treatment of *H. pylori* by oxidative stress and competitive acid inhibitor mechanisms, the influence of gastrointestinal flora on *H. pylori*, probiotic adjuvant therapy of *H. pylori*-related GU, and the immunoprotective effect of neutrophil activator protein could be popular research directions and trends in the future.

## Introduction

Gastric ulcer (GU) is one of the most common diseases affecting humans. An unbalance in defense factors and detrimental factors is the primary cause of GU, which is characterized by rhythmic epigastric pain after eating (1, 2). *Helicobacter pylori* (*H. pylori*) is a Gram-negative bacterium that selectively colonizes the epithelium of the gastric mucosa (3). It is a common bacterial infection in the world and infects more than 50% of the world’s population (4). Approximately, 75% of GUs are caused by *H. pylori* (5). It is known that *H. pylori* is a carcinogen, and *H. pylori*-related GUs have also been linked to gastric cancer (6, 7). An infection caused by *H. pylori* must be eradicated medically, as the bacteria relapse easily. Bismuth, antibiotics, and the inhibition of gastric acid secretion are usually used to treat GUs caused by *H. pylori* (8–10). *H. pylori* has become more resistant to antibiotics, making eradication increasingly difficult (11). Nowadays, GU caused by *H. pylori* infection has become an important issue. Therefore, it is necessary to conduct a statistical analysis of the research status, hotspots, and trends of *H. pylori*-related GUs.

In bibliometric analysis, the literature is identified, developmental trends are clarified both objectively and visibly, the current status is determined, and the future development of the field is predicted (12). Bibliometrics is widely used in oncology, ophthalmology, complementary medicine, alternative medicine, and other interdisciplinary fields of medicine (13, 14). However, bibliometric studies on *H. pylori*-related GU are still absent. In this study, we conducted bibliometric research on *H. pylori*-related GU papers that were published from 2012 to 2022 and analyzed the research status, hotspots, and trends in the field. This study will hopefully provide valuable information and aid in future research on GU-related *H. pylori*.

A major objective of this article was to review the current research status and hotspots of *H. pylori*-related GU and to propose research directions for the future. By using bibliometric analysis software, such as VOSviewer and CiteSpace, these questions were addressed through the following goals: (a) the number and timing of publications; (b) journals, institutions, and countries of publications; (c) domains of study; (d) citation and co-citation of publications; and (e) keywords used in publications.

## Methods

### Criteria for inclusion and exclusion

The papers were screened according to the Preferred Reporting Items for Systematic Reviews and Meta-Analyses (PRISMA) guidelines (15). The study included articles that met the following criteria: *H. pylori*-related GU research, including clinical, therapeutic drugs, risk factors, pathogenesis, and prognosis; and articles published in English due to their higher recognition. If an article met one or more of the following criteria, it was excluded: non-English text, retracted publication, letter, meeting abstract, editorial material, and articles irrelevant to the *H. pylori*-related GU.

### Data sources and search strategy

The data were retrieved from the Web of Science Core Collection (WoSCC). The following was the search strategy: [TS = (gastric ulcer) or GU] and [TS = HP or (*Helicobacter pylori*) or *H. pylori*] and (Language = English) and (Document type = Article or Review), Publication date from “2012-01-01” to “2022-07-17.”

### Data screening

Articles were screened by two independent reviewers according to the inclusion and exclusion criteria. If any disagreement between the two reviewers is observed, a discussion is held with the third reviewer to decide whether to include the article. Search results were exported into a plain text file with a record content of “Full Record and Cited References,” saved as “download_*.txt.”

### Data analysis and tools

The number of publications (Nps) was used to measure the output capacity of an author, institution, or country in a field. The number of citations (Ncs) and the global citations score (GCS) were used to determine the growth of scientific impact (16). The H-index was designed to assess the academic contributions of researchers, affiliations, and countries and predict future scientific accomplishments (17). Furthermore, the impact factor (IF) and the journal citation report (JCR) were used to measure the quality and impact of medical journals (18).

The bibliometric mapping and clustering analysis were conducted using VOSviewer, which builds and visualizes bibliometric networks. Nodes represent publications, and lines show the strength of the relationship. By assigning nodes to different clusters, the same color indicates the nodes belonging to the same cluster and have a high correlation between them (19). Co-authorship, co-occurrences, and co-citations analysis were performed with the help of VOSviewer 1.6.18 in this study.

A bibliometric tool, CiteSpace, identifies trends and hotspots in the publications and enables users to explore areas of expertise and emerging research topics within knowledge areas (20). In this study, CiteSpace 6.1.R2 Basic software was used to perform keywords citation bursts, references, dual-map, and timeline views.

Bioinformatics (an online platform^1^) was used to generate pictures, and Excel was used for data statistics and tabulation.

## Results

**FIGURE 1:**
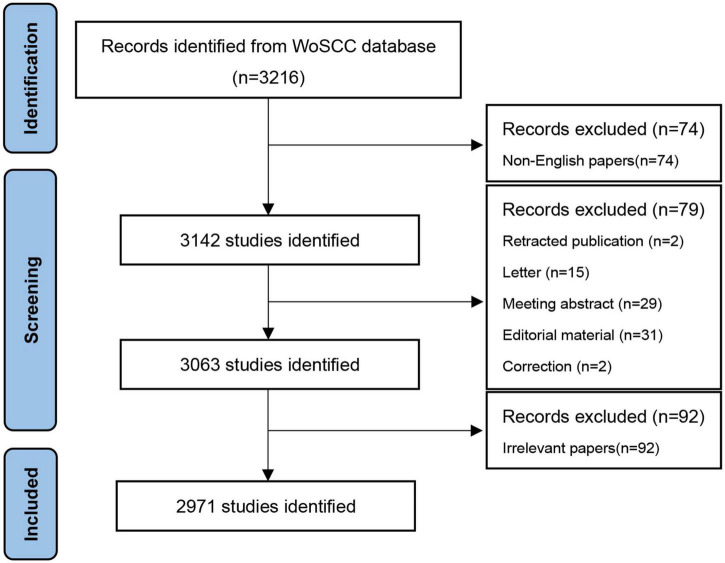
Retrieval strategies from the Web of Science Core Collection (WoSCC) and inclusion criteria for *Helicobacter pylori*-related gastric ulcer (GU) articles.

There were 3,216 potentially relevant records (last searched on 17 July 2022). Figure 1 illustrates the selection process according to PRISMA 2020. Bibliometric analyses were performed on 2,971 studies that met the inclusion criteria.

### Trends in the global publishing

**FIGURE 2:**
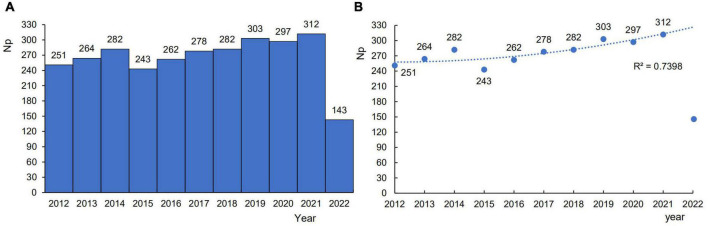
The global trends in publications on *Helicobacter pylori*-related gastric ulcer (GU). **(A)** An overview of global publications growth between 2012 and 2022 is shown in the histogram. **(B)** The curve shows the fit of the model to growth trends in publications.

The global trends in publications on *H. pylori*-related GU remained at a high level and showed a fluctuating upward trend in Figure 2. The most articles were published in 2021. It suggested that *H. pylori*-related GU is a major concern for researchers.

### Distribution of countries/regions for paper publication

**FIGURE 3:**
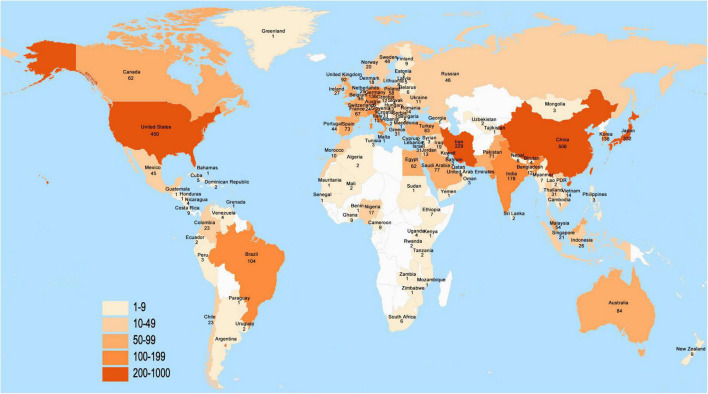
The distribution of *Helicobacter pylori*-related gastric ulcer (GU) research worldwide, from 2012 to 2022.

**FIGURE 4:**
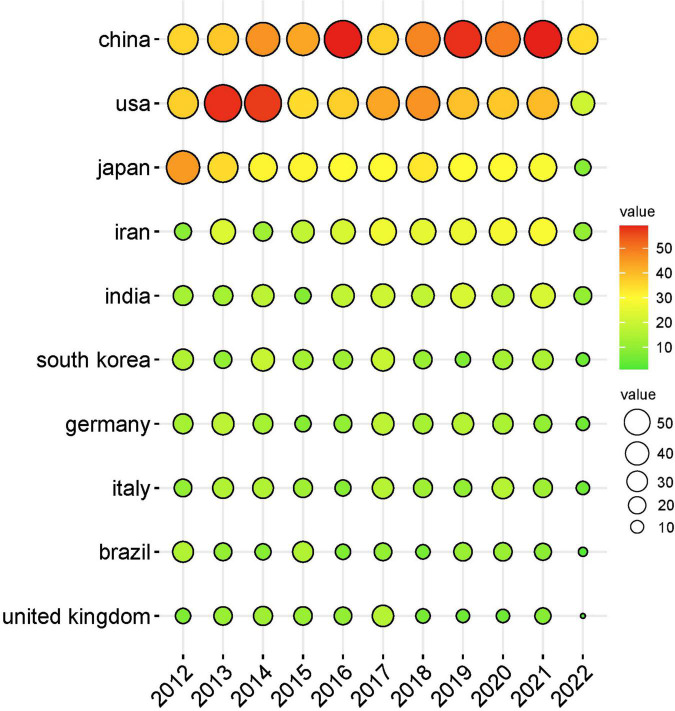
Top 10 countries/regions of annual publications on *Helicobacter pylori*-related gastric ulcer (GU) research, from 2012 to 2022. A country’s number of articles issued is directly proportional to the size of the circle and the color of the circle from green to red.

A total of 115 countries/regions around the world contributed to the research on *H. pylori*-related GU, and the number of publications issued by countries/regions is shown in Figure 3. The top 10 countries/regions of all publications are shown in Table 1. It is clear that China was the most productive country with 508 publications (17.10%), followed by the United States with 450 publications (15.15%), and Japan with 322 publications (11.17%). In terms of total citations, the articles of the United States were referenced the most with 16,434 times, followed by China with 8,237 times, Japan with 7,491 times, and Germany with 7,322 times. Furthermore, the United States had the highest H-index (53), followed by Japan (41), China (34), Germany (33), and Italy (30). The annual publication of papers in the top 10 countries/regions is shown in Figure 4.

**TABLE 1:** Top 10 high-output countries/regions in the field of *Helicobacter pylori*-related gastric ulcer (GU).

Rank	Country/ Region	Np	Percent of 2,971 publications	Nc	H-index
1	China	508	17.10%	8,237	34
2	United States	450	15.15%	16,434	53
3	Japan	332	11.17%	7,491	41
4	Iran	229	7.71%	2,639	28
5	India	178	6.00%	2,627	22
6	South Korea	138	4.64%	1,751	22
7	Germany	136	4.58%	7,322	33
8	Italy	131	4.41%	5,340	30
9	Brazil	104	3.50%	1,565	23
10	United Kingdom	92	3.10%	6,246	29

### Analysis of institutions

As shown in Table 2, the top 10 productive affiliations around the world were listed. Baylor College of Medicine contributed the max number of papers, followed by Oita University and Tehran University of Medical Sciences. Meanwhile, Baylor College of Medicine was also the institution with the highest number of citations (3,690 times) and H-index (249), followed by Michael E. DeBakey VA Medical Center and Oita University, which suggested that those affiliations had a high influence on this field. Of these institutions, three were from the United States and three were from Iran. It indicated that more affiliations in the United States and Iran are looking at *H. pylori*-related GU research.

**TABLE 2:** Top 10 institutions with the most publications in *Helicobacter pylori*-related gastric ulcer (GU).

Rank	Institution	Country	*p*	Nc	H-index
1	Baylor College of Medicine	United States	68	3,690	249
2	Oita University	Japan	57	1,399	196
3	Tehran University of Medical Sciences	Iran	49	935	82
4	Michael E. DeBakey VA Medical Center	United States	41	2,556	169
5	Vanderbilt University	United States	37	933	80
6	China Medical University	China	33	586	78
7	Islamic Azad University	Iran	33	342	43
8	Seoul National University	South Korea	32	594	84
9	Shahid Beheshti University Medical Sciences	Iran	27	380	41
10	University of Malaya	Malaysia	26	577	43

### Analysis of journals

**FIGURE 5:**
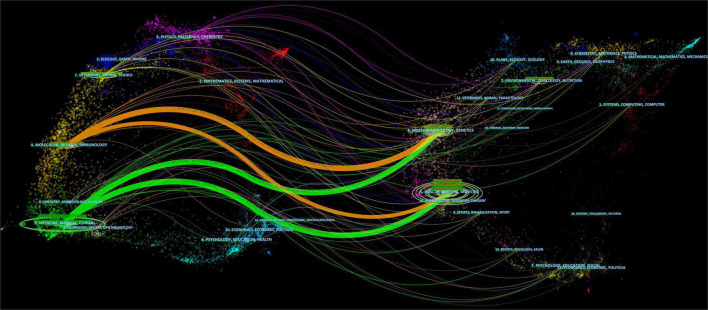
The dual-map overlay of journals in the field of *Helicobacter pylori*-related gastric ulcer (GU). Citing journals appear on the left, cited journals appear on the right, and citation relationships are represented by colored paths.

**FIGURE 6:**
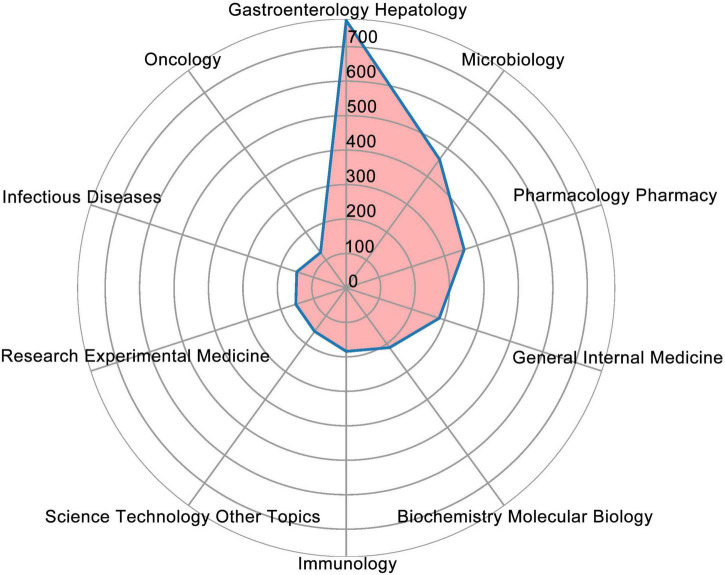
Top 10 research directions for the journals of *Helicobacter pylori*-related gastric ulcer (GU).

In Table 3, the top 10 journals for *H. pylori*-related GU publications are listed. *World Journal of Gastroenterology* was the most productive journal with 129 publications, which was followed by *Helicobacter* (113) and *PLOS One* (78). As shown in Table 4, although *GUT* (IF: 31.793) published only 13 articles, it had the highest Nc in the field of *H. pylori*-related GU. While *Gastroenterology* and *the American Journal of Gastroenterology* had only a few articles, their citations were very high. A great deal of impact was caused by *H. pylori* on the field of GU in these journals. Besides, the association between cited and co-cited journals was analyzed using a dual-map overlay (Figure 5). The wiring between the left and right nodes describes the citation path and illustrates the connections between different research areas. There were four critical lines on the map, and it showed that papers published in molecular/biology/immunology and medicine/medical/clinical field usually cite articles in molecular/biology/genetics or health/nursing/medicine fields. Figure 6 describes the top 10 research directions of journals in the field of *H. pylori*-related GU; gastroenterology and hepatology had the largest number of publications with 776 articles, followed by microbiology with 461 articles, and pharmacology and pharmacy with 360 articles.

**TABLE 3:** Top 10 journals with the most publications in the field of *Helicobacter pylori*-related gastric ulcer (GU).

Rank	Journal	Np	Nc	JCR (2021)	IF (2021)
1	World Journal of Gastroenterology	129	3,713	Q2	5.374
2	Helicobacter	113	1,827	Q2	5.182
3	PLOS One	78	1,665	Q2	3.752
4	BMC Gastroenterology	34	400	Q4	2.847
5	Scientific Reports	34	375	Q2	4.996
6	Microbial Pathogenesis	33	565	Q3	3.848
7	Digestive Diseases and Sciences	32	440	Q3	3.487
8	Journal of Ethnopharmacology	32	620	Q1/Q2	5.195
9	Journal of Gastroenterology and Hepatology	26	416	Q2	4.369
10	Frontiers in Microbiology	25	293	Q1	6.064

**TABLE 4:** Top 10 journals with the most citations in the field of *Helicobacter pylori*-related gastric ulcer (GU).

Rank	Journal	Np	Nc	JCR (2021)	IF (2021)
1	GUT	13	4,234	Q1	31.793
2	World Journal of Gastroenterology	129	3,713	Q2	5.374
3	Helicobacter	113	1,827	Q2	5.182
4	PLOS One	78	1,665	Q2	3.752
5	Gastroenterology	5	1,338	Q1	33.883
6	American Journal of Gastroenterology	8	1,057	Q1	12.045
7	Alimentary Pharmacology and Therapeutics	19	938	Q1	9.524
8	Journal of Ethnopharmacology	32	620	Q1/Q2	5.195
9	Microbial Pathogenesis	33	565	Q3	3.848
10	Journal of Gastroenterology	17	550	Q2	6.772

### Analysis of authors

Table 5 shows that the Japanese authors made up two of the top three. Yamaoka Y from Oita University in Japan was the most productive author in the field of *H. pylori*-related GU with 47 articles (H-index: 21), next was Graham DY from Baylor College of Medicine in the United States with 30 articles (H-index: 21), and Sugimoto M from Tokyo Medical University in Japan with 20 articles (H-index: 10). Graham DY had the highest number of citations (with 4,536 times), followed by Malfertheiner P from University Medical Center Hamburg-Eppendorf in Germany with 3,698 times and Yamaoka Y with 1,024 times. It indicated that the studies of Graham DY, Malfertheiner P, and Yamaoka Y have attracted more attention from researchers.

**TABLE 5:** Top 10 authors with the most publications in the field of *Helicobacter pylori*-related gastric ulcer (GU).

Rank	Author	Country	Institution	Np	Nc	H-index
1	Yamaoka Y	Japan	Oita University	47	1,024	21
2	Graham DY	USA	Baylor College of Medicine	30	4,536	21
3	Sugimoto M	Japan	Tokyo Medical University	20	299	10
4	Malfertheiner P	Germany	University Medical Center Hamburg-Eppendorf	18	3,698	13
5	Shiota S	Japan	Oita University	16	525	12
6	Vadivelu J	Malaysia	University of Malaya	16	416	11
7	Bagheri N	Iran	Shahrekord University of Medical Sciences	16	367	11
8	Miftahussurur M	Indonesia	Airlangga University	16	215	8
9	Backert S	Germany	University of Erlangen Nuremberg	15	598	12
10	Rahimian G	Iran	Shahrekord University of Medical Sciences	15	336	10

### Analysis of references

**FIGURE 7:**
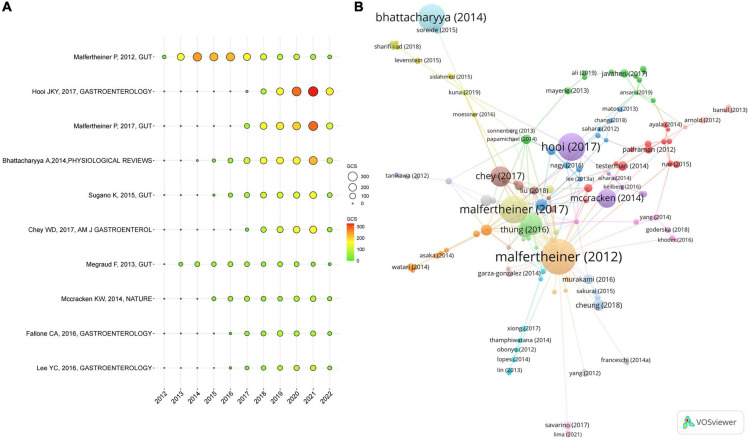
Citations for the references. **(A)** The top 10 cited papers in GCS. **(B)** The network map of the document citation.

Figure 7A displays the yearly number of GCS for the top 10 cited papers with a high GCS ranging between 2012 and 2022. The article with the highest GCS was written by Malfertheiner P published in 2012 in the *GUT*, followed by the article written by Hooi JKY published in 2017 in the *Gastroenterology*, and the article written by Malfertheiner P published in 2017 in the *GUT*. The network of articles with high citation frequencies is shown in Figure 7B.

**FIGURE 8:**
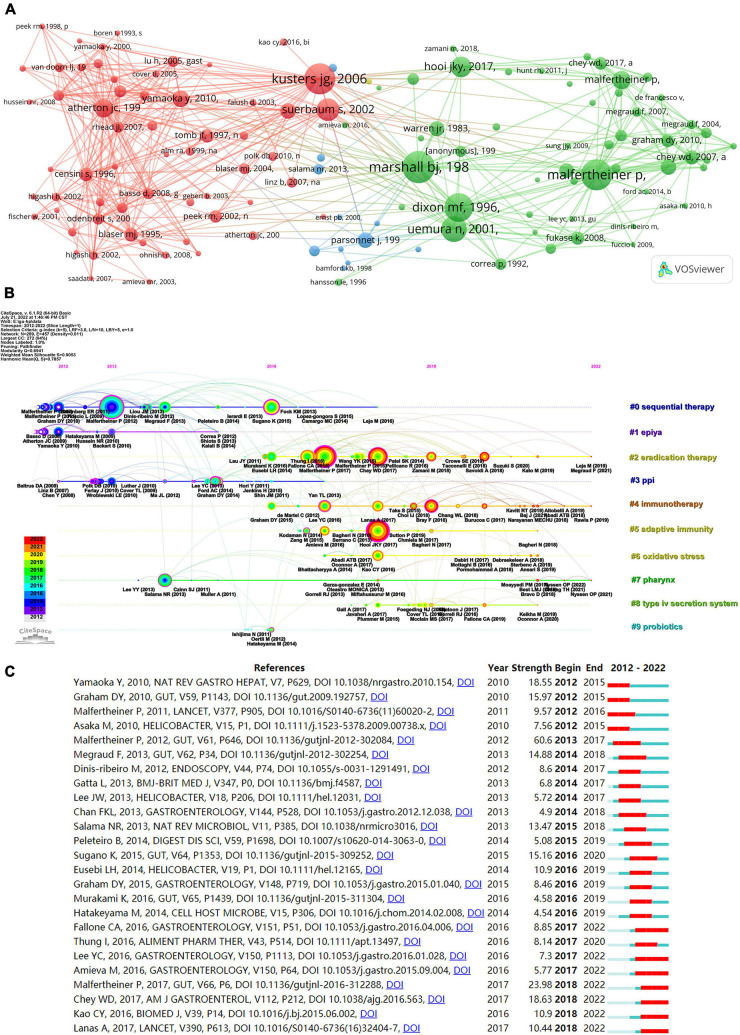
The co-cited references map on *Helicobacter pylori*-related gastric ulcer (GU). **(A)** The network for co-cited references. **(B)** The timeline view for co-cited references. **(C)** The top 25 co-cited references with the strongest citation bursts.

The co-citation of cited references is presented in Figure 8A. In total, 125 references (co-cited in more than 40 citations) were analyzed by VOSviewer. Nodes connected by lines indicate that they were cited in the same publication, while a shorter line indicates a closer relationship. Citations for the top three references were as follows: the largest number of co-citation references was written by Marshall BJ published in 1984 in the *Lancet* (288 times), followed by the article written by Malfertheiner P published in 2012 in the *GUT* (277 times), and the article written by Kusters JG published in 2006 in the *Clinical Microbiology Reviews* (274 times). Figure 8B shows the 10 major clusters of co-cited references, namely, sequential therapy, epiya, eradication therapy, PPI, immunotherapy, adaptive immunity, oxidative stress, pharynx, type IV secretion system, and probiotics. Cites bursts refer to references that have been cited frequently over a long period. The top 25 references with the strongest citation bursts are shown in Figure 8C. Among the results of burst strength, Malfertheiner P (21) had the highest burst strength of 60.6. In addition, Amieva (22), Malfertheiner (23), Chey (24), Kao (25), and Lanas (26) received more attention.

### Analysis of keywords

**FIGURE 9:**
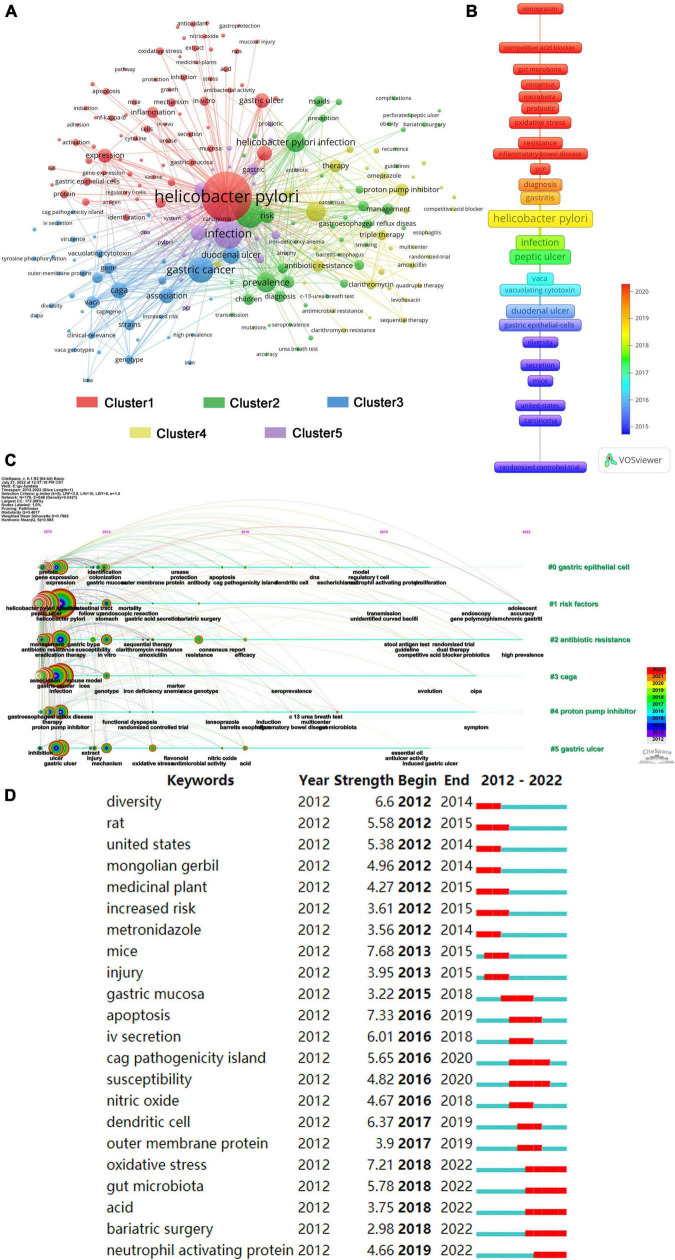
Keywords map for *Helicobacter pylori*-related gastric ulcer (GU). **(A)** The keyword co-occurrence network that displays the clustering of keywords through different colors, and the same color indicates that these keywords were in the same cluster. **(B)** The graph of the average year in which keywords appear, with red keywords appearing later than dark blue. **(C)** Keyword clustering timeline graph. **(D)** Top 22 keywords with the most bursts.

Keywords reflect the core content of the paper. The network visualization of the keywords is shown in Figure 9A. The larger the node, the more frequently the keyword appears. The nodes of the same color were in the same cluster. Cluster 1 (red) was mainly concerned with expression level, inflammation, pathway, and mechanism. Cluster 2 (green) chiefly focused on *H. pylori* infection, prevalence, and management. Cluster 3 (blue) was primarily concerned with gene expression. Therapy and resistance were the main concerns of cluster 4 (yellow). Cluster 5 (purple) mainly focused on the disease related to *H. pylori* infection. The average year of these keywords is shown in Figure 9B, and the keywords colored in red represented a later year of appearance. As can be seen from the figure, oxidative stress, microbiota, probiotics, competitive acid blocker, and vonoprazan were popular topics in recent years. Furthermore, as shown in Figure 9C, gastric epithelial cells, risk factors, antibiotic resistance, cage, proton pump inhibitor, and gastric ulcer have been a long focus of research in *H. pylori*-related GU. Figure 9D shows the most popular topics in the recent 5 years including oxidative stress, gut microbiota, acid, bariatric surgery, and neutrophil-activating protein.

## Discussion

In this article, the publications related to *H. pylori*-related GU were collected through the WoSCC database. The bibliometric software, such as VOSviewer and CiteSpace, were used to visually analyze the temporal and spatial distribution of literature, keywords, and the citations and co-citations of publications. Additionally, VOSviewer and CiteSpace were used to clarify the research status, hotspots, and trends of *H. pylori*-related GU. This article aimed to provide a reference for researchers in the field of gastrointestinal diseases.

### Research status of *Helicobacter pylori*-related gastric ulcer

In total, 2,971 articles were included in our analysis. *H. pylori*-related GU papers showed an increasing trend between 2012 and 2022, and researchers have been paying close attention to it. Among the 115 countries that have published a paper on *H. pylori*-related GU, China was certainly the most productive country, followed by the United States, and Japan. The largest number of publications in China indicated that *H. pylori*-related GU has a high incidence rate in China, which may be related to the eating habits of the Chinese. In terms of affiliations, Baylor College of Medicine had the highest number of publications, citations, and H-index. Three of the top five agencies were from the United States, indicating that the United States has a strong academic ability in the field.

In the field of *H. pylori*-related GU, the United States has the highest citation and H-index, indicating it has the greatest influence. This apparent superiority might be attributed to the leading academics with advanced research facilities and technologies. Although China has the highest number of publications, the number of citations was close to Japan and Germany. This means that Germany and Japan had a higher quality of research on *H. pylori*-related GU, and Chinese researchers should improve the quality of their work.

An article with a high IF is considered to be of higher quality when it appears in a journal. The journals were mainly in the gastroenterology and hepatology, microbiology, and pharmacology and pharmacy fields. In the field of *H. pylori*-related GU, fewer articles were published in journals with high IF. The *World Journal of Gastroenterology* published the most in *H. pylori*-related GU studies, and it was also the second most cited journal. There is no doubt that this journal played a crucial role in studying *H. pylori*-related GU. Remarkably, the *GUT* ranked first in terms of citations with 13 publications, and the *Gastroenterology* ranked fifth with five publications. Researchers interested in this field should pay closer attention to these journals since the articles in these journals were of high quality.

The most productive author, Yamaoka Y, has published 47 papers on *H. pylori*-related GU fields and received 1,024 citations. Yamaoka Y mainly studies the virulence factors of *H. pylori* and its mechanism of action (27). Based on the author’s highest-cited article, there are differences in virulence factors of *H. pylori* in different regions, and the pathogenicity of *H. pylori* is correlated with virulence factors (28). In addition to being the second most cited author, Graham DY from the United States was also the author with the largest citation. Graham DY focuses primarily on the epidemiology and treatment of *H. pylori*-associated ulcers, and in one of his highest-cited articles, he followed up patients with ulcers who participated in randomized controlled trials to cure ulcers for 2 years and found that triple therapy could reduce ulcer recurrence rates and that most peptic ulcers associated with *H. pylori* infection were curable (29). In another highly cited paper, Graham DY pointed out that the effectiveness of clarithromycin-containing triple therapy in the treatment of *H. pylori* was reduced and proposed treatment suggestions and follow-up clinical experimental research directions for this condition, which provided great help for the treatment and clinical research of *H. pylori* (30).

### Research hotspots and trends

Articles with high citations can reflect the issues of concern in a research field. The papers written by Malfertheiner P published in the *GUT* in 2012 and 2017 were ranked first and third in GCS. In those two Consensus Reports, the author and experts made recommendations for the prevention, diagnosis, and treatment of *H. pylori*, which provided support for clinicians in the treatment of *H. pylori*-related disease (21, 23). A paper written by Hooi JKY published in *Gastroenterology* in 2017 ranked second. Through the systematic review and meta-analysis, the authors found that there were differences in infection rates in different countries/regions and that more than half of the world’s population was infected with *H. pylori* (31). It suggested that the prevention and treatment of *H. pylori* have always been a hot issue in this field.

The keywords and co-cited references timeline view can reflect hotspots in the field of *H. pylori*-related GU research. It can be seen from the co-cited references timeline view that eradication therapy and immunotherapy were the common concerns in *H. pylori*-related GU. Lim JH and his team found that the efficacy of levofloxacin-based 3-line eradication therapy for *H. pylori*-associated peptic ulcers was similar to that of first-/second-line eradication therapies (32). Fahimi F and his team showed the importance of VacA in the pathogenesis of *H. pylori*-related diseases and discussed the potential and possibility of VacA as a targeted immunotherapy for the treatment of *H. pylori* (33). In the keyword clustering timeline view, risk factors and antibiotic resistance are more concerning to researchers. Harris RB and his team studied *H. pylori* infection in the Three Navajo Nation Chapter Communities and found that *H. pylori* infection in the region was associated with households and the use of uncontrolled water sources (34). Le LTT determined that the *H. pylori* resistance rates were 80.6% for clarithromycin, 71.7% for amoxicillin, 49.4% for metronidazole, 45.1% for levofloxacin, and 11.4% for tetracycline, especially in children who had previously received *H. pylori* eradication treatment (35).

As *H. pylori*-related GU disease research continues to progress, the research hotspot is changing as well. Co-cited references with the most bursts, keyword average year view, and emergence map show hot issues and directions in current research. The cited literature studies in the past 5 years have highlighted the main research directions for *H. pylori*-related GU and are listed as follows: the treatment of antibiotic-resistant *H. pylori*-related Gus (23, 24), the pathogenesis of *H. pylori* (25), and the prevention and treatment of complications caused by anti- *H. pylori* therapy (26). Among the keywords mentioned more frequently over the past few years were oxidative stress, probiotics, competitive acid blocker, vonoprazan, gut microbiota, and neutrophil-activating protein. Vonoprazan is a competitive acid blocker that inhibits gastric acid secretion by binding competitively to the proton pump with potassium ions (36). The study by Ashida et al. (37) and his team showed that vonoprazan-based triple therapy is safe and effective. Through a review, Butcher LD discussed how oxidative stress can cause *H. pylori* infections. Based on oxidative stress mechanisms, Silvan JM and his team explored a treatment method for *H. pylori* infection (38, 39). Studies have shown that the fixed value of *H. pylori* is related to the gastric microbiota, which shows a decrease in gastric microbial diversity and an increase in the relative abundance of bacteria (40, 41). In the research conducted by Devi et al. (42), bifidobacterium was found to be associated with *H. pylori* infection and to protect against *H. pylori*-associated stomach disorders. Research by Zhao et al. (43) found that neutrophil-activating proteins are responsible for promoting H_2_O_2_-induced biofilm formation in bacteria, which makes them multidrug resistant. Based on these keywords, it can be seen that *H. pylori*-associated GU research is focused on pathogenesis and treatment. There is no doubt that future studies will continue to focus on *H. pylori* pathogenesis and the efficacy and safety of therapeutic drugs.

## Strengths and limitations

In this article, CiteSpace and VOSviewer were used to conduct a comprehensive bibliometric analysis of *H. pylori*-related GU, and the results were objective and reliable. It provided an overview of the status, trends, and hotspots in *H. pylori*-related GU research. However, there are still some limitations to the research presented in this article. Only the WoSCC database was analyzed, and only English publications were included, which may lead to selection bias.

## Conclusion

Through bibliometric research, the global publications of *H. pylori*-related GU are on the rise, and the disease is receiving widespread attention. The findings of the study indicated that China published the most articles, while the United States was the most influential country. The pathogenesis and treatment of *H. pylori*-related GU have always been hot topics of research. Research on oxidative stress mechanisms, competitive acid blocker, and neutrophil-activating protein may be at the forefront of future research. Besides the traditional antibiotic-based triple and quadruple therapies, other treatments can also be studied, such as natural medicine preparations. Essentially, this article provides a scientific and quantitative analysis of *H. pylori*-related GU research from a global perspective and suggests future research directions.

## Author contributions

CY analyzed the data and drafted the manuscript. CO and MZ carried out a literature search. JQ and MX drew the figures. HS reviewed and revised the manuscript. All authors produced the results of this study and gave final approval to the version submitted.

## Footnotes

1
http://www.bioinformatics.com.cn/

## References

[1] Brucker MC Faucher MA. Pharmacologic management of common gastrointestinal health problems in women. *J Nurse Midwifery.* (1997) 42:145–62. 10.1016/S0091-2182(97)00031-1 9239966

[2] Yang RQ Mao H Huang LY Su PZ Lu M. Effects of hydrotalcite combined with esomeprazole on gastric ulcer healing quality: A clinical observation study. *World J Gastroenterol.* (2017) 23:1268–77. 10.3748/wjg.v23.i7.1268 28275307 PMC5323452

[3] Radin JN González-Rivera C Ivie SE McClain MS Cover TL. *Helicobacter* pylori VacA induces programmed necrosis in gastric epithelial cells. *Infect Immun.* (2011) 79:2535–43. 10.1128/IAI.01370-10 21482684 PMC3191986

[4] Fukase K Kato M Kikuchi S Inoue K Uemura N Okamoto S Effect of eradication of *Helicobacter* pylori on incidence of metachronous gastric carcinoma after endoscopic resection of early gastric cancer: an open-label, randomised controlled trial. *Lancet.* (2008) 372:392–7. 10.1016/S0140-6736(08)61159-9 18675689

[5] Tveit AH Bruce MG Bruden DL Morris J Reasonover A Hurlburt DA Alaska sentinel surveillance study of *Helicobacter pylori* isolates from Alaska Native persons from 2000 to 2008. *J Clin Microbiol.* (2011) 49:3638–43. 10.1128/JCM.01067-11 21813726 PMC3187320

[6] Vogiatzi P Cassone M Luzzi I Lucchetti C Otvos L Jr. Giordano A. *Helicobacter pylori* as a class I carcinogen: physiopathology and management strategies. *J Cell Biochem.* (2007) 102:264–73. 10.1002/jcb.21375 17486575

[7] Feng J Guo J Wang JP Chai BF. MiR-32-5p aggravates intestinal epithelial cell injury in pediatric enteritis induced by *Helicobacter pylori*. *World J Gastroenterol.* (2019) 25:6222–37. 10.3748/wjg.v25.i41.6222 31749593 PMC6848013

[8] Greenberg ER Anderson GL Morgan DR Torres J Chey WD Bravo LE 14-day triple, 5-day concomitant, and 10-day sequential therapies for *Helicobacter pylori* infection in seven Latin American sites: a randomised trial. *Lancet.* (2011) 378:507–14. 10.1016/S0140-6736(11)60825-8 21777974 PMC3313469

[9] Niu M Zhou Y Xie Y Li X Tian Y Yao L Comparison of the dual therapy of ilaprazole-amoxicillin and the bismuth quadruple therapy of ilaprazole-amoxicillin-furazolidone-bismuth glycyrrhizinate for eradication of *Helicobacter pylori*. *Front Pharmacol.* (2022) 13:771876. 10.3389/fphar.2022.771876 35571120 PMC9094360

[10] Kim YS Lee JH Song J Kim H. Gastroprotective effects of inulae flos on HCl/Ethanol-Induced Gastric Ulcers in Rats. *Molecules.* (2020) 25:5623. 10.3390/molecules25235623 33260419 PMC7730672

[11] Burkitt MD Duckworth CA Williams JM Pritchard DM. *Helicobacter pylori*-induced gastric pathology: insights from in vivo and ex vivo models. *Dis Model Mech.* (2017) 10:89–104. 10.1242/dmm.027649 28151409 PMC5312008

[12] Brandt JS Hadaya O Schuster M Rosen T Sauer MV Ananth CV. A bibliometric analysis of top-cited journal articles in obstetrics and gynecology. *JAMA Netw Open.* (2019) 2:e1918007. 10.1001/jamanetworkopen.2019.18007 31860106 PMC6991228

[13] Qi X Li Y Liu W Wang Y Chen Z Lin L. Research trend of publications concerning antibody-drug conjugate in solid cancer: A bibliometric study. *Front Pharmacol.* (2022) 13:921385. 10.3389/fphar.2022.921385 35795565 PMC9252465

[14] Zhang J Song L Jia J Tian W Lai R Zhang Z Knowledge mapping of necroptosis from 2012 to 2021: A bibliometric analysis. *Front Immunol.* (2022) 13:917155. 10.3389/fimmu.2022.917155 35769473 PMC9234124

[15] Page MJ McKenzie JE Bossuyt PM Boutron I Hoffmann TC Mulrow CD The PRISMA 2020 statement: an updated guideline for reporting systematic reviews. *BMJ.* (2021) 372:n71. 10.1136/bmj.n71 33782057 PMC8005924

[16] Deng P Wang S Sun X Qi Y Ma Z Pan X Global trends in research of gouty arthritis over past decade: A bibliometric analysis. *Front Immunol.* (2022) 13:910400. 10.3389/fimmu.2022.910400 35757713 PMC9229989

[17] Noruzi A Gholampour B Gholampour S Jafari S Farshid R Stanek A Current and future perspectives on the COVID-19 Vaccine: A scientometric review. *J Clin Med.* (2022) 11:750. 10.3390/jcm11030750 35160202 PMC8836413

[18] Roldan-Valadez E Salazar-Ruiz SY Ibarra-Contreras R Rios C. Current concepts on bibliometrics: a brief review about impact factor, Eigenfactor score, CiteScore, SCImago Journal Rank, Source-Normalised Impact per Paper, H-index, and alternative metrics. *Ir J Med Sci.* (2019) 188:939–51. 10.1007/s11845-018-1936-5 30511320

[19] van Eck NJ Waltman L. Software survey: VOSviewer, a computer program for bibliometric mapping. *Scientometrics.* (2010) 84:523–38. 10.1007/s11192-009-0146-3 20585380 PMC2883932

[20] Chen C Chen Y. Searching for clinical evidence in CiteSpace. *AMIA Annu Symp Proc.* (2005) 2005:121–5. 16779014 PMC1560638

[21] Malfertheiner P Megraud F O’Morain CA Atherton J Axon AT Bazzoli F Management of *Helicobacter pylori* infection–the Maastricht IV/Florence Consensus Report. *Gut.* (2012) 61:646–64. 10.1136/gutjnl-2012-302084 22491499

[22] Amieva M Peek RM Jr. Pathobiology of *Helicobacter pylori*-induced gastric cancer. *Gastroenterology* (2016) 150:64–78. 10.1053/j.gastro.2015.09.004 26385073 PMC4691563

[23] Malfertheiner P Megraud F O’Morain CA Gisbert JP Kuipers EJ Axon AT Management of *Helicobacter pylori* infection-the Maastricht V/Florence Consensus Report. *Gut.* (2017) 66:6–30. 10.1136/gutjnl-2016-312288 27707777

[24] Chey WD Leontiadis GI Howden CW Moss SF. ACG clinical guideline: Treatment of *Helicobacter pylori* infection. *Am J Gastroenterol.* (2017) 112:212–39. 10.1038/ajg.2016.563 28071659

[25] Kao CY Sheu BS Wu JJ. *Helicobacter pylori* infection: An overview of bacterial virulence factors and pathogenesis. *Biomed J.* (2016) 39:14–23. 10.1016/j.bj.2015.06.002 27105595 PMC6138426

[26] Lanas A Chan FKL. Peptic ulcer disease. *Lancet.* (2017) 390:613–24. 10.1016/S0140-6736(16)32404-7 28242110

[27] Yamaoka Y Graham DY. *Helicobacter pylori* virulence and cancer pathogenesis. *Future Oncol.* (2014) 10:1487–500. 10.2217/fon.14.29 25052757 PMC4197059

[28] Yamaoka Y. Mechanisms of disease: *Helicobacter pylori* virulence factors. *Nat Rev Gastroenterol Hepatol.* (2010) 7:629–41. 10.1038/nrgastro.2010.154 20938460 PMC3137895

[29] Graham DY Lew GM Klein PD Evans DG Evans DJ Jr. Saeed ZA Effect of treatment of *Helicobacter pylori* infection on the long-term recurrence of gastric or duodenal ulcer. A randomized, controlled study. *Ann Intern Med.* (1992) 116:705–8. 10.7326/0003-4819-116-9-705 1558340

[30] Graham DY Fischbach L. *Helicobacter pylori* treatment in the era of increasing antibiotic resistance. *Gut.* (2010) 59:1143–53. 10.1136/gut.2009.192757 20525969

[31] Hooi JKY Lai WY Ng WK Suen MMY Underwood FE Tanyingoh D Global prevalence of *Helicobacter pylori* infection: Systematic review and meta-analysis. *Gastroenterology* (2017) 153:420–9. 10.1053/j.gastro.2017.04.022 28456631

[32] Lim JH Kim SG Song JH Hwang JJ Lee DH Han JP Efficacy of levofloxacin-based third-line therapy for the eradication of *Helicobacter pylori* in peptic ulcer disease. *Gut Liver.* (2017) 11:226–31. 10.5009/gnl16099 27609487 PMC5347646

[33] Fahimi F Tohidkia MR Fouladi M Aghabeygi R Samadi N Omidi Y. Pleiotropic cytotoxicity of VacA toxin in host cells and its impact on immunotherapy. *Bioimpacts.* (2017) 7:59–71. 10.15171/bi.2017.08 28546954 PMC5439391

[34] Harris RB Brown HE Begay RL Sanderson PR Chief C Monroy FP *Helicobacter pylori* prevalence and risk factors in three rural indigenous communities of northern arizona. *Int J Environ Res Public Health.* (2022) 19:797. 10.3390/ijerph19020797 35055622 PMC8775467

[35] Le LTT Nguyen TA Nguyen NA Nguyen YTH Nguyen HTB Nguyen LT Antibiotic resistance of *Helicobacter pylori* in children with gastritis and peptic ulcers in mekong delta, vietnam. *Healthcare (Basel).* (2022) 10:1121. 10.3390/healthcare10061121 35742177 PMC9222858

[36] Abadi ATB Ierardi E. Vonoprazan and *Helicobacter pylori* Treatment: A lesson from japan or a limited geographic phenomenon? *Front Pharmacol.* (2019) 10:316. 10.3389/fphar.2019.00316 31024299 PMC6459936

[37] Ashida K Honda Y Sanada K Takemura Y Sakamoto S. The safety and effectiveness of vonoprazan-based *Helicobacter pylori* eradication therapy; a prospective post-marketing surveillance. *Expert Opin Drug Saf.* (2019) 18:1255–61. 10.1080/14740338.2019.1676722 31646920

[38] Butcher LD den Hartog G Ernst PB Crowe SE. Oxidative stress resulting from *Helicobacter pylori* infection contributes to gastric carcinogenesis. *Cell Mol Gastroenterol Hepatol.* (2017) 3:316–22. 10.1016/j.jcmgh.2017.02.002 28462373 PMC5404027

[39] Silvan JM Gutierrez-Docio A Guerrero-Hurtado E Domingo-Serrano L Blanco-Suarez A Prodanov M Pre-Treatment with grape seed extract reduces inflammatory response and oxidative stress induced by *Helicobacter pylori* infection in human gastric epithelial cells. *Antioxidants (Basel).* (2021) 10:943. 10.3390/antiox10060943 34208004 PMC8230724

[40] Das A Pereira V Saxena S Ghosh TS Anbumani D Bag S Gastric microbiome of Indian patients with *Helicobacter pylori* infection, and their interaction networks. *Sci Rep.* (2017) 7:15438. 10.1038/s41598-017-15510-6 29133866 PMC5684312

[41] Noto JM Peek RM Jr. The gastric microbiome, its interaction with *Helicobacter pylori*, and its potential role in the progression to stomach cancer. *PLoS Pathog.* (2017) 13:e1006573. 10.1371/journal.ppat.1006573 28982167 PMC5629027

[42] Devi TB Devadas K George M Gandhimathi A Chouhan D Retnakumar RJ Low bifidobacterium abundance in the lower gut microbiota is associated with *Helicobacter pylori*-Related Gastric Ulcer and Gastric Cancer. *Front Microbiol.* (2021) 12:631140. 10.3389/fmicb.2021.631140 33717022 PMC7953064

[43] Zhao Y Cai Y Chen Z Li H Xu Z Li W SpoT-mediated NapA upregulation promotes oxidative stress-induced *Helicobacter pylori* biofilm formation and confers multidrug resistance. *Antimicrob Agents Chemother.* (2021) 65:e152–121. 10.1128/AAC.00152-21 33649116 PMC8092859

